# Pandemic (H1N1) 2009 Vaccination and Class Suspensions after Outbreaks, Taipei City, Taiwan

**DOI:** 10.3201/eid1608.100310

**Published:** 2010-08

**Authors:** Po-Ren Hsueh, Ping-Ing Lee, Allen Wen-Hsiang Chiu, Muh-Yong Yen

**Affiliations:** National Taiwan University Hospital, Taipei, Taiwan (P.-R. Hsueh, P.-I. Lee); Taipei City Hospital, Taipei (A. Wen-Hsiang Chiu, M.-Y. Yen)

**Keywords:** Pandemic (H1N1) 2009, vaccination, influenza virus, class suspensions, Taipei City, Taiwan, dispatch

## Abstract

In Taipei City, class suspensions were implemented beginning September 1, 2009 when transmission of pandemic (H1N1) 2009 infection was suspected. The uptake rate of pandemic (H1N1) 2009 vaccination (starting on November 16, 2009) among students 7–18 years of age was 74.7%. Outbreaks were mitigated after late November 2009.

As of April 25, 2010, >214 countries have reported laboratory confirmed cases of pandemic (H1N1) 2009, including >17,919 deaths. Preparedness measures such as having substantial antiviral drug stockpiles for treatment and chemoprophylaxis and implementation of vaccination programs are considered crucial for effective control of the pandemic (*1*,*2*). In many countries, vaccination programs for pandemic (H1N1) 2009 were implemented by the end of 2009.

The US Centers for Disease Control and Prevention (CDC) recommends that nonpharmaceutical interventions should be implemented to reduce influenza transmission between persons after an outbreak and before vaccination programs begin (*3*). During the 1918–19 and 2009 influenza pandemics, class suspension and school closures, either reactively following outbreaks or proactively at a regional level, were implemented by many countries (*4**–**8*). CDC guidance suggests that during an influenza outbreak, policymakers should weigh the advantages and disadvantages of school closures before making a decision (*9*).

## The Study

**Figure 1:**
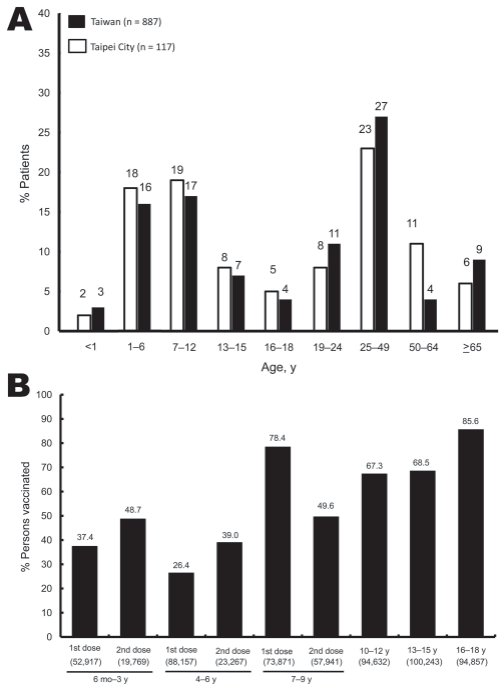
A) Incidence of hospitalization among patients infected with pandemic (H1N1) 2009 in each age group in Taipei City and throughout Taiwan as of week 4, 2010. B) Pandemic (H1N1) 2009 vaccine coverage among persons <18 years of age by age group in Taipei City as of week 4, 2010 (from November 11, 2009, to January 29, 2010). Numbers within parentheses indicate the number of persons within each age group who were recommended for vaccination.

In Taiwan, persons were confirmed to have pandemic (H1N1) 2009 infection if they had an acute febrile respiratory illness with an epidemiologic link and a positive test result for pandemic (H1N1) 2009. From June 1, 2009, through January 29, 2010, a total of 3,159 patients having documented pandemic (H1N1) 2009 infections were reported to the Taiwan Centers for Disease Control (Taiwan CDC) (*10*). Of these patients, 887 (28.1%) were hospitalized and 39 (4.4%) died (Figure 1, panel A). In Taipei City, 117 hospitalized patients infected with pandemic (H1N1) 2009 virus were reported, and 4 patients (3.4%) died during the same period. Patients <18 years of age accounted for 51.9% and 47.9% of all hospitalized patients in Taiwan and Taipei City, respectively (Figure 1, panel A) (*10*).

In Taiwan, a 2-3-5 intervention policy for class suspension was implemented beginning September 1 (week 35 of 2009) for all students <18 years of age when transmission of influenza-like illness or influenza A/B virus infection (identified by positive rapid antigen test [RAT] for influenza A/B) was suspected (*11*). Three commercial kits were available: QuickVue Influenza A+B (Quidel Corporation, San Diego, CA, USA), BD Directogen EZ Flu A+B (Becton Dickinson, Sparks, MD, USA), and BinaxNOW Influenza A&B (BinaxNOW, Portland, ME, USA) (*11*). Under the 2-3-5 policy, any class was suspended if >2 students were noted to have a positive RAT result or confirmed pandemic (H1N1) 2009 within 3 days. Students from suspended classes were asked to stay home for at least 5 days before returning to school**.** In addition, students’ temperatures were checked at the entrance of each school during the pandemic; if any student’s temperature was >38^o^C, the student was suspended.

Two pandemic (H1N1) 2009 vaccines were available: Focetria (Novartis, Basel, Switzerland, available since November 1, 2009) and AdimFlu-S (Adimmune Corporation, Taichung, Taiwan, available since November 16, 2009). Vaccination of front-line healthcare personnel began November 2, 2009; infants >6 months and <1 year received vaccination beginning November 11, 2009. Beginning November 16, 2009, pregnant women, preschool children 1–6 years of age and students 7–12 years of age were vaccinated. Students 13–15 years of age (since November 23, 2009) and 16–18 years of age (since November 30, 2009) (Figure 1, panel B), and persons with major illness or injury were vaccinated simultaneously (*10*). For children 6 months to 9 years of age, a second dose of vaccine was recommended with an interval of at least 3 weeks (*10*). For this age group, coverage rate of the vaccine in this report included those who had received at least 1 dose of vaccine. Immunization with pandemic (H1N1) 2009 vaccines was free and voluntary.

By January 29, 2010, less than half (5.6 million) of the planned doses (12 million) of pandemic (H1N1) 2009 vaccine had been administered. The overall coverage of the vaccine in Taiwan (population ≈23 million**)** was ≈24.3%; the rate was 21.8% for Taipei City (population 2.6 million). Coverage rates of pandemic (H1N1) 2009 vaccine for different age groups as of January 29, 2010, in Taipei City are shown in Figure 1, panel B. Overall, the coverage rate of the vaccine for school students 7–18 years of age was 271,460/363,603 (74.7%) as of January 29, 2010. The coverage rate for younger children (<6 years of age) and persons 19–24 years of age was 30.3% (first dose) and 3.1%, respectively (*10*).

**Figure 2:**
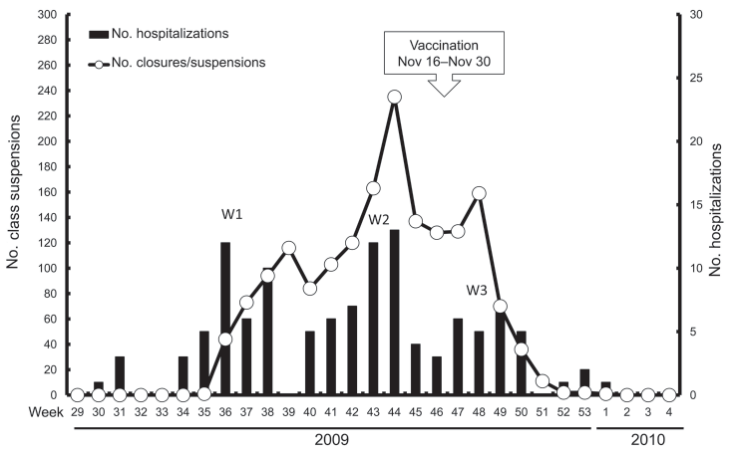
Weekly number of class suspensions (including nursery schools, kindergartens, elementary/primary schools, and junior and senior high schools) and new hospitalized patients caused by pandemic (H1N1) 2009, confirmed by real-time reverse transcription–PCR in Taipei City, Taiwan, from week 29 in 2009 to week 4 in 2010. W1–3, 3 waves of pandemic (H1N1) 2009 outbreaks. See text for details of the vaccination program for pandemic (H1N1) 2009 for school children 7–18 years of age.

As of January 29, 2010, a total of 1,708 classes in Taipei City’s elementary/primary, junior, and senior high schools had been suspended (Figure 2). The peak number of class suspensions (without school closures) occurred during September and November 2009, and no class was suspended after week 4 of 2010 (*10*).

In Taipei City, a total of 171 hospitalized patients infected with influenza viruses were identified from June 1, 2009, through January 29, 2010. These included 117 hospitalized patients infected with pandemic (H1N1) 2009 (Figure 2) (*11*). The pandemic (H1N1) 2009–related death rates were 1.54 and 1.78 per 1 million population in Taipei City and Taiwan, respectively; the death rate for Taiwan was the third lowest among the 32 members and observers of the Organization for Economic Cooperation and Development (*12*). Three waves of influenza were identified with activities that paralleled the intensity of class suspensions (Figure 2).

## Conclusions

Although only about one fifth of the population in Taipei City had received pandemic (H1N1) 2009 vaccination, the number of hospitalized patients with pandemic (H1N1) 2009 declined remarkably after mid-November to December 2009; no cases were reported after January 29, 2010. The rationale of the 2-3-5 intervention policy in Taiwan was based on the incubation period of seasonal influenza. If influenza developed in 2 students in the same class within 3 days, it was anticipated that the virus had already been spread within the class. Because influenza virus shedding begins 24 hours before illness onset, we assumed that a 5-day observation period should detect all infected classmates.

The classroom structure in Taiwan’s middle schools and high schools is different from western countries and referred to as a “platoon” system. A group of students are placed together in a specific homeroom with a core teacher who also provides counseling to students and performs administrative work. Other teachers who specialize in different subjects move from class to class for teaching. The core teacher and administrative officials can audit off-school activities of each student through information technology.

Class suspensions or school closures alone may not be able to quell an epidemic, but these nonpharmaceutical interventions may be able to provide additional time to prepare for vaccination (*5**–**8*). Children**,** especially older students in middle or high school, play a primary role in transmission of influenza within schools, families, and communities and should be a key target group for vaccination (*11*,*13*,*14*). In our study, the number of class suspensions also decreased concurrently with the declining trend of hospitalized patients with pandemic (H1N1) 2009. Implementation of a vaccination policy for students, which began in mid-November (week 47), resulted in a remarkable decline of the third wave of pandemic (H1N1) 2009 two weeks later (week 49). This scenario suggests that the high vaccine coverage rate among students 6–18 years of age, as well as the 2-3-5 intervention policy, might have contributed to the rapid mitigation and subsequent cessation of the outbreak.

The results of our study demonstrate a more effective mitigation strategy to control influenza outbreaks during the wait for vaccines. Citywide class suspensions in Taipei City and the high uptake rate of vaccination among students may have had a combined effect in ending the influenza outbreaks.
